# Identification of Diaryl-Quinoline Compounds as Entry Inhibitors of Ebola Virus

**DOI:** 10.3390/v10120678

**Published:** 2018-11-30

**Authors:** Qinghua Cui, Han Cheng, Rui Xiong, Gang Zhang, Ruikun Du, Manu Anantpadma, Robert A. Davey, Lijun Rong

**Affiliations:** 1 College of Pharmacy, Shandong University of Traditional Chinese Medicine, Jinan 250355, China; ruikun7128@gmail.com; 2 Department of Microbiology and Immunology, College of Medicine, The University of Illinois at Chicago, Chicago, IL 60612, USA; hancheng@uic.edu; 3 Department of Medicinal Chemistry and Pharmacognosy, College of Pharmacy, and UICentre, University of Illinois at Chicago, Chicago, IL 60612, USA; rxiong3@uic.edu; 4 State Key Laboratory of Bioactive Substances and Function of Natural Medicine, Institute of Materia Medica, Peking Union Medical College and Chinese Academy of Medical Sciences, Beijing 100050, China; gzhang@imm.ac.cn; 5 Department of Virology and Immunology, Texas Biomedical Research Institute, San Antonio, TX 78227, USA; manantpadma@txbiomed.org (M.A.); radavey@bu.edu (R.A.D.); 6 Department of Microbiology, Boston University, National Emerging Infectious Diseases Laboratories, 401P, 620 Albany Street, Boston, MA 02118, USA

**Keywords:** EBOV, entry inhibitor, quinoline, glycoprotein, lead compound, assay

## Abstract

Ebola virus is the causative agent of Ebola virus disease in humans. The lethality of Ebola virus infection is about 50%, supporting the urgent need to develop anti-Ebola drugs. Glycoprotein (GP) is the only surface protein of the Ebola virus, which is functionally critical for the virus to attach and enter the host cells, and is a promising target for anti-Ebola virus drug development. In this study, using the recombinant HIV-1/Ebola pseudovirus platform we previously established, we evaluated a small molecule library containing various quinoline compounds for anti-Ebola virus entry inhibitors. Some of the quinoline compounds specifically inhibited the entry of the Ebola virus. Among them, compound SYL1712 was the most potent Ebola virus entry inhibitor with an IC_50_ of ~1 μM. The binding of SYL1712 to the vial glycoprotein was computationally modeled and was predicted to interact with specific residues of GP. We used the time of the addition assay to show that compound SYL1712 blocks Ebola GP-mediated entry. Finally, consistent with being an Ebola virus entry inhibitor, compound SYL1712 inhibited infectious Ebola virus replication in tissue culture under biosafety level 4 containment, with an IC_50_ of 2 μM. In conclusion, we identified several related molecules with a diaryl-quinoline scaffold as potential anti-EBOV entry inhibitors, which can be further optimized for anti-Ebola drug development.

## 1. Introduction

The species Zaire Ebola virus, a member of the genus Ebola virus in the family *Filoviridae* is considered a significant public health concern due its high fatality rate. In humans, the infection can cause Ebola virus disease (EVD), which is a lethal acute hemorrhagic disease [1]. In the past 40 years, more than 30 Ebola virus disease outbreaks in African countries have been recorded. In 2013–2016, a large-scale epidemic broke out in Western Africa, and more than 10,000 deaths were confirmed. The most recent EVD epidemic was reported in the Democratic Republic of the Congo in 2018, which was first reported in May [2].

The development of anti-EBOV agents has been hampered partly due to the biosafety level 4 (BSL-4) containment requirement to handle the infectious Ebola virus. Three types of anti-Ebola agents have been reported as potential prophylactics and/or therapies against Ebola virus infection: (1) vaccines or antibodies: a few potential vaccines and monoclonal antibody cocktail “Zmapp” have been shown to be highly effective in protecting non-human primates against lethal Ebola virus infections [3,4]; (2) small molecule inhibitors either targeting the viral proteins including RNA polymerase inhibitors (favipiravir and remdesivir) [5,6], VP35 protein inhibitor (GA017) [7], and glycoprotein [8,9] or host proteins [10,11,12]; and (3) different nucleic acids, such as siRNA [13] and antisense morpholine substituted oligonucleotides [14], were reported as potential anti-Ebola agents.

**Figure 1:**
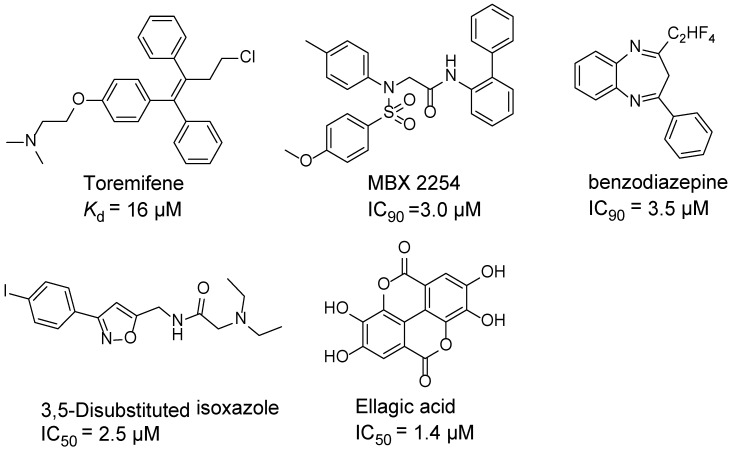
Small molecule inhibitors targeting Ebola glycoprotein.

Ebola virus glycoprotein (GP) is the only viral surface protein and is solely responsible for receptor binding and mediating fusion of viral and host membranes during viral entry [15,16]. GP is composed of a heterodimer of GP_1_ and GP_2_ that forms a trimer. GP is considered a promising target for anti-Ebola drug development [8], as inhibitors could block viral entry into cells, which is the first step of virus replication. Antibody-based therapies work by blocking this step [17,18]. A few inhibitors, as listed in Figure 1, have been identified to have anti-EBOV activity, possibly via targeting GP, but none have been approved for therapeutics in humans or in clinical trials [9,19,20,21].

To identify and develop new anti-EBOV agents, we evaluated a small library of diaryl-quinoline compounds (Supplementary Table S1), which were previously shown to have anti-tuberculosis activity [22], for their anti-Ebola virus entry ability, and we found that some of these compounds are potent entry inhibitors against Ebola virus entry, and as a result, subsequent viral replication.

## 2. Materials and Methods

### 2.1. Cell Culture

Human lung epithelial cell line A549 (ATCC#CCL185, Manassas, VA, USA), human embryonic kidney cell line 239T (ATCC# CRL-1573, Manassas, VA, USA), African green monkey (Vero) cells (ATCC #CRL-1586, Manassas, VA, USA), and HeLa cell line (ATCC#CCl-2, Manassas, VA, USA) were cultured in Dulbecco modified Eagle medium (DMEM, Cellgro, Manassas, VA, USA) supplemented with 10% fetal bovine serum (FBS, Gibco, Carlsbad, CA, USA), 100 µg/mL streptomycin, and 100 units of penicillin (Invitrogen, Carlsbad, CA, USA) at 37 °C and 5% CO_2_.

### 2.2. Generation of Pseudovirions

Three types of recombinant pseudoviruses (HIV-1/EBOV, HIV-1/H5N1, and HIV-1/LASV) were used in this study, and they all contained the corresponding viral membrane glycoproteins (GPs). The plasmids containing GP-encoding genes used were: influenza A virus HA, NA, from A/Goose/Qinghai/59/05 (H5N1) [23]; LASV-GP [24]; EBOV-GP; and HIV-1 core plasmid pNL4-3.Luc.R-E- [25]. HIV-1/EBOV, HIV-1/H5N1, and HIV-1/LASV plasmids were transiently transfected into 293T cells of 50–70% confluency using a polyethylenimine-based transfection protocol. After 6 h, the cells were washed with PBS buffer and replaced with 20 mL clear medium in a culture dish (150 mm). The supernatant was collected 24 h later, passed through 0.45 µm Millipore filter (Nalgene, Rochester, NY, USA), and stocked at 4 °C for use.

### 2.3. Collection of Compounds

We obtained a small library of 92 quinoline compounds with potent antitubercular activities from Dr. Huaqing Cui (Peking Union Medical College and Chinese Academy of Medical Sciences, Beijing, China). The structural information of these compounds is provided in the Supplementary Table S1. These compounds were dissolved in DMSO and stored at a stock concentration of 10 mM.

### 2.4. High-Throughput Screen of Anti-EBOV Inhibitor

A549 cells were seeded in 384-well plates (1000 cells per well) one day before infection with HIV-1/EBOV, HIV-1/H5N1, or HIV-1/LASV pseudoviruses. The compounds were added into the wells together with the viruses at a final concentration of 10 μM in 0.0625% DMSO, and the plates were incubated for 48 h. The luciferase activity in the infected cells was quantified by a Neolite reporter gene detection system (PerkinElmer, Waltham, MA, USA, Cat# 6016719). The wells treated with viruses alone with 0.0625% DMSO were used as negative controls, and the wells treated with viruses together with an HIV reverse transcriptase inhibitor azidothymidine (AZT, 5 μM) were used as positive controls. Each compound was tested in duplicate.

Cell viability measurement of A549 cells was performed 48 h after treatment of compounds or DMSO control in DMEM supplemented with 10% FBS, using the CellTiter-Glo kit (Promega, Madison, WI, USA). The hit compounds were two-fold serially diluted from 100 μM (total 8 concentrations) for dose-response titration, and the CC_50_s and IC_50_s were calculated with Prism software (7.04 Edition, GraphPad Software, La Jolla, CA, USA). The assays were performed with 2 replicate wells and repeated twice.

### 2.5. Computation Study

We used a similar docking method as reported in our previous publication [26]. Briefly, the GLIDE module (Schrödinger 10.0, Portland, OR, USA) within Schrödinger Suite 2014 was used to rank the best conformations and orientations of the ligand based on its interactions with the Ebola virus GP (PDB code, 5JQ7) The LigPrep (including Ionizer) module and Macromodel module was used to generate three-dimensional coordinates of the ligands and protein with calculated ionization states. A grid file (20 Å by 20 Å) centered on toremifene in the cavity of GP was produced. Docking conformational flexibility of the ligands was handled via an exhaustive conformational search. Top-scored conformations was visualized using UCSF CHIMERA (version 1.12, University of California San Francisco, San Francisco, CA, USA).

### 2.6. Time-of-Addition Experiment

A549 cells (96-well plates, 10,000 cells per well) were incubated with HIV-1/EBOV pseudovirions for 1 h at 4 °C, and the supernatant was removed from the wells and the cells were washed twice with PBS buffer. Fresh DMEM was added into the cells, and cells were then incubated at 37 °C in CO_2_ incubator. At different time points, compounds were added with the final concentration of 10 μM. The experiment was performed in triplicate wells using benztropine (25 μM) as a control. Virus infection was quantified by luciferase signals 48 h after infection. For each time point, the data were normalized by the values from the DMSO vehicle-treated control wells.

### 2.7. Infectious Virus Assays

Infectious Ebola virus assays were performed in the biosafety level 4 (BSL-4) facility at the Texas Biomedical Research Institute, USA. The infectious EBOV was produced as previously described [27]. The EBOV Mayinga isolate (EBOV-GFP, GenBank: KF990213.1) used in this assay was from Heinz Feldmann (NIH, Rocky Mountain Laboratory, Hamilton, MT, USA), which contains an insertion of green fluorescent protein (GFP) between the nucleoprotein (NP) and VP35 [28]. The virus was grown in Vero cells for 7 days, and the culture supernatants containing the viruses were centrifuged through a 20% sucrose cushion (141,118× *g* for 2 h) at 4 °C. The virus pellets were suspended in PBS buffer, and stored in aliquots at −80 °C until use.

For infection assays, HeLa cells were first seeded in 384-well tissue culture plates (4000 cells per well) and cultured overnight in DMEM medium with 10% FBS. Next, the cells were infected by EBOV-GFP virus (MOI = 0.05 to 0.15) in the presence of the test compounds at various concentrations. Bafilomycin (10 μM) was used as the positive control. All the concentrations were tested in duplicate. After 24 h incubation, post-infection cells were fixed by adding formalin at 4 °C. After 24 h fixation, formalin was removed, and the plates were washed twice with PBS buffer. Then, cell nuclei were stained with Hoechst dyes (Cat number: 62249, Thermo Fisher Scientific, Waltham, MA, USA). Blue fluorescent cell nuclei (blue) and green fluorescent infected cells expressing GFP can be imaged under fluorescent microscopy. The numbers of cell nuclei and infected cells were counted using CellProfiler software version 2.1.1 (Broad Institute, Cambridge, MA, USA). A total number of nuclei (blue) was used as a check of cell function, with >30% reduction, indicating a growth arrest or toxicity, which were noted during analysis.

## 3. Results

### 3.1. Diaryl-Quinoline Compounds Specifically Block the Entry of Ebola Virus

Although there is still no FDA approved therapy for human use, several anti-EBOV drugs are being developed [11,29,30]. Research shows that a few anti-microbial agents, including anti-malarial, were also reported with reasonable antiviral activities [31]. Bedaquiline is a FDA approved second line anti-tuberculosis drug, which is specifically used to treat multi-drug-resistant tuberculosis (MDR-TB). In this study, we screened a small library of bedaquiline derivatives for potential novel Ebola virus entry inhibitors using a pseudotyped (HIV-1/EBOV) entry assay [32]. Three types of pseudoviruses (HIV-1/EBOV, HIV-1/H5N1, and HIV-1/LASV) were simultaneously used in the initial screen to reduce false positives and to identify Ebola-specific entry inhibitors. Many quinoline compounds showed strong inhibition against EBOV entry (Supplemental Table S1) at 10 µM. Then, compounds with anti-EBOV activity (specific inhibition >80%, host cell viability >70%) were selected for further evaluation and the results are listed in Table 1. These compounds did not inhibit the entry of either HIV-1/H5N1 or HIV-1/LASV, whereas they displayed inhibition of Ebola GP-mediated entry (more than 80% at a final concentration of 10 µM), and were non-toxic to the cells (cell viability >90%, at the same concentration).

The IC_50_s of the 10 selected compounds against the HIV-1/EBOV pseudovirus entry were determined, and they ranged from 0.95 to 8.65 μM (Table 2). The CC_50_ of these compounds were in the range of 109.5 to 241.9 μM, indicating that these compounds were not toxic to the host cells (A549). The calculated SI (CC_50_/ IC_50_) values of these compounds were from 12.7 to 225.9. Compound SYL1712 was the most potent inhibitor among these compounds, with an IC_50_ of 0.95 μM and an SI value of 225.9.

### 3.2. Time-of-Addition (TOA) Experiments

**Figure 2:**
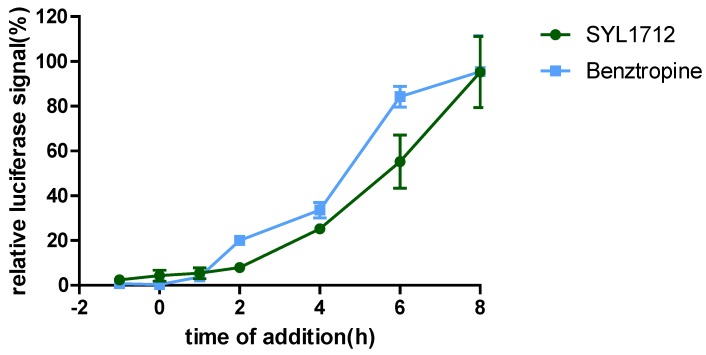
SYL1712 inhibits EBOV infection at late entry step. Pseudotyped HIV-1/EBOV was incubated with A549 cells at 4 °C at the one-hour time point. After one hour of incubation, the pseudovirus was removed and temperature was shifted to 37 °C to trigger virus internalization. SYL1712 (10 µM) and benztropine (25 µM) were introduced at different time points of virus infection, and the compounds’ effects on viral infection are shown (means ± SD; *n* = 3). The luciferase signals were normalized based on the signals from the vehicle DMSO-treated wells at each time point. Relative luciferase signal at 100% means the drug has no effect on the EBOV entry at each time point.

To explore the mechanism of action (MOA) of compound SYL1712 in inhibiting Ebola virus entry, time-of addition (TOA) experiments were performed following a previously published protocol using benztropine as a positive control, which was reported as an EBOV entry inhibitor by interfering with the GP-mediated fusion between virus membrane and host endosomal membrane [33,34]. The results (Figure 2) show that both benztropine and compound SYL1712 similarly blocked viral entry well within one hour, and gradually lost the activity afterward, suggesting that compound SYL1712, like benztropine, blocks Ebola GP-mediated viral entry in the endosome.

### 3.3. Compound SYL1712 Inhibits Replication of Infectious EBOV

**Figure 3:**
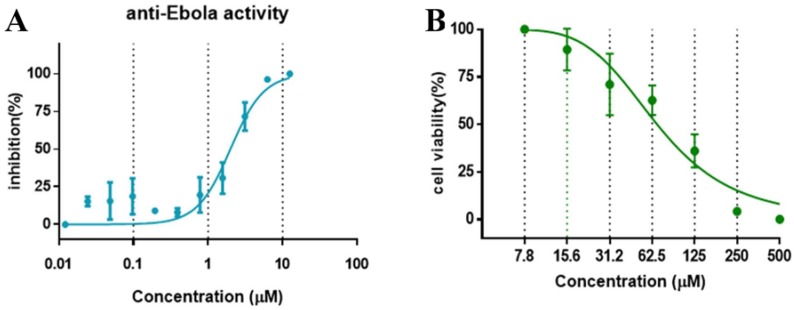
The in vitro dose-response curves of SYL1712 are shown against (**A**) EBOV/Mayinga infections and (**B**) in cell toxicity assay in HeLa cells. Data are means ± SD (*n* = 3) from three independent experiments.

The anti-virus effect of compound SYL1712 was further evaluated and validated in HeLa cells against infectious EBOV infection in a BSL-4 facility. The high-content imaging detection method with Ebola Mayinga isolate was used for this study [27]. Compound SYL1712 was effective at blocking the replication of infectious EBOV with an IC_50_ of 1.99 μM (Figure 3A), consistent with the results from the pseudovirus entry assay above. Since the CC_50_ of compound SYL1712 was 74.08 μM with HeLa cells (Figure 3B), the selective index (SI) of compound SYL1712 was 37.2.

### 3.4. Structure-Activity Relationship and Docking Studies of the Diaryquinoline Compounds

**Figure 4:**
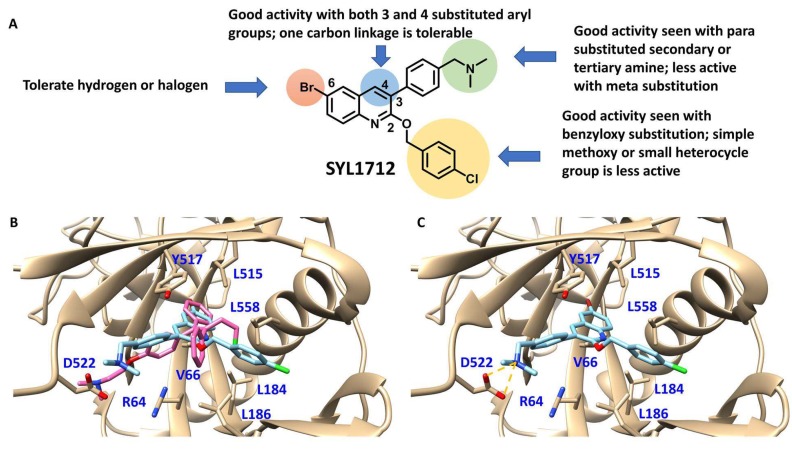
Structure activity relationship (SAR) of diarylquinoline compounds against EBOV (**A**) Summary of the SAR analysis the diarylquinoline compounds as Ebola virus entry inhibitors. (**B**,**C**) Predicted binding mode of SYL1712 with Ebola glycoprotein by molecular docking (PDB code: 5JQ7). (**B**) Superposition of SYL1712 (cyan) with toremifene (pink) shows similar binding orientations at the interface between GP1 and GP2; (**C**) SYL1712 (cyan) forms both hydrophobic interactions (V66, Y517, L558, L184 and L186) and electrostatic interactions (D522) with EBOV GP (**C**).

Although there is still no FDA approved therapy for human use, several classes of anti-EBOV drugs are in preclinical or clinical development including but not limited to: antihistamines, antimuscarinics, estrogen receptor antagonists, calcium-channel blockers, topical anesthetics, and selective serotonin reuptake inhibitors [11,30,31]. In this study, we screened a small library of bedaquiline (an FDA approved second line anti-tuberculosis drug) derivatives for identification of potential novel Ebola virus entry inhibitors using a pseudotyped (HIV-1/EBOV) entry assay. The library contains 92 bedaquiline analogs from the in-house library of Dr. Huaqing Cui (Peking Union Medical College and Chinese Academy of Medical Sciences, Beijing, China). The structure-activity relationship (SAR) analysis of diaryquinoline analogs (Supplemental Table S1) is summarized in Figure 4A, which highlights the key substitutions on the quinoline core. Most active compounds contain terminal basic amine side chains, exemplified by SYL1712 that bears a (dimethylamino)methyl)phenyl chain. Compounds SYL1642 and SYL1658, with less basic amine chain (amide or pyrazol, predicted pKa < 3), were less potent against EBOV, suggesting the pKa of the terminal chain is essential for an active inhibitor. 2-benzyloxy substitution (SYL1658, SYL168, or SYL1712) showed better potency compared to heterocycle (SYL1711) or simple methoxy groups (SYL1640, SYL1642, SYL1654, SYL1655, or SYL1660). Aryl substitution on the 3- or 4-position of quinoline is required for good anti-EBOV activity. The 6-position of quinoline tolerates simple hydrogen or a halogen group from our screening; additional evaluation is needed to dissect the role of this region for EBOV activity.

We further evaluated the binding position of SYL1712 in toremifene binding pocket at EBOV GP using molecular docking. The optimized conformation of compound SYL1712 highly overlapped with toremifene and is predicted to engage with similar residues in the binding pocket (Figure 4B) [35]. The 3-phenyl-quinoline core forms a parallel-displaced π-stacking interaction with Y517 (Figure 4C), explaining the enhanced activity of this chemical scaffold against EBOV. The terminal nitrogen N(CH_3_)_2_ on the tail of compound SYL1712 forms a strong electrostatic interaction with D522 within 3.5 Å, consistent with our SAR analysis on the terminal basic amine side chain. The 2-benzyloxy chain extends out from the ethyl chloride pocket of toremifene to interact with L184 and L186 for an extra hydrophobic interaction. This region has been shown to interact with ligands in newer published crystal structures (e.g., structure of EBOV GP in complex with benztropine; PDB code: 6F6S) [33]. Overall, SYL1712 is predicted to highly fit the toremifene-binding pocket, potentially explaining the good potency of this compound.

## 4. Discussion

In this study, we used an HIV-1-based pseudotyped virus screening platform to evaluate a small library of diarylquinoline compounds, and we identified a number of Ebola virus-specific entry inhibitors. The most potent inhibitor of this series of compounds, SYL1712, has an IC_50_ of 1 μM using the pseudotyped virus, and an IC_50_ of 2 μM using infectious Ebola virus. The time-of-addition (TOA) experiment and computational docking study support the notion that this compound likely binds to the same cavity of Ebola GP, as do toremifene and other small molecule inhibitors [36], and blocks the GP-mediated entry. The structure-activity relationship (SAR) analysis suggests medicinal chemistry options to further improve the potency of this series of inhibitors. Therefore, we think that this scaffold can be further optimized for developing anti-EBOV therapeutics. In addition to anti-EBOV activity, SYL1712 also inhibited LASV entry, though not as dramatically as what we observed with EBOV. This suggests SYL1712 might also affect endosomal trafficking, which could be investigated for its potential as a broad-spectrum antiviral via modulating endosomal trafficking in the future.

Numerous small molecule therapeutic candidates that directly target the EBOV GP or critical host factors for viral entry have been reported. Most of these inhibitors target events occurring in the endosome, such as GP proteolytic processing, endosomal trafficking, interactions with NPC1, and fusion, including: (1) CA-074, FY-DMK, CID23631927, K11777, and cathepsin B and L inhibitors [29,30,37,38,39,40,41]; (2) inhibitors targeting host factors in the late endosomal steps in EBOV entry, such as U18666A, MBX2254, and MBX2270, targeting NPC1 [19,20,42], and numerous L-type calcium channel inhibitors, including verapamil, tetrandrine, nimodipinee, and diltiazem [10]; (3), a group of selective estrogen receptor modulators (SERMs), such as clomiphene and toremifene, which can directly bind to Ebola GP [36,43]; and (4) numerous G-protein coupled receptor (GPCR) antagonists such as antihistamines, targeting Ebola virus (and Marburg virus) GPs [34,44]. Only the first generation of antihistamines, such as diphenhydramine and chlorcyclizine, effectively block entry of Ebola virus and Marburg viruses, consistent with the notion that these compounds exert their antiviral effect in the endosomes [26].

A number of SERMS, such as toremifene, and GPCR antagonists, such as benztropine, were shown to bind to a pocket at the GP1/GP2 subunit interface of the Ebola GP [33,34,35,36]. Analysis of this binding pocket provides insight into the mechanism by which such diverse scaffolds of small molecules inhibit entry of Ebola virus. This insight could be used for rational drug design to improve the potency of these inhibitors. As shown above, compound SYL1712, although structurally different from the SERMS and GPCR antagonists, could bind to the same pocket on the Ebola GP demonstrated via docking analysis (Figure 4B,C), suggesting a similar mechanism of inhibition. Measuring the interaction between protein and small molecules with low molecular weight can help the evaluation of the candidate compounds, but our choices of the biophysical methods are limited. So far, only a thermal shift assay has been applied to measure the interaction, but this assay needs Ebola glycoprotein in its natural conformation, which we currently were not able to obtain. However, the preliminary SAR analysis of compound SYL1712 and its analogs (Figure 4A) revealed a path forward on the further optimization of the inhibitors for potential drug development.

## Figures and Tables

**Table 1:** List of 10 selected quinoline compounds with specific EBOV entry inhibition at 10 µM.

ID	Structure	Inhibition			Cell Viability
		HIV-1/H5N1	HIV-1/LASV	HIV-1/EBOV	
SYL1640	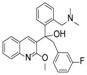	−1.97%	−15.08%	79.06%	97.64%
SYL1642	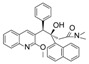	14.11%	34.99%	92.15%	72.30%
SYL1654	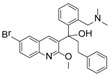	8.04%	18.79%	84.84%	94.74%
SYL1655	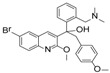	−17.17%	28.87%	90.29%	95.31%
SYL1660	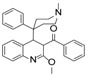	3.34%	−26.55%	97.48%	106.02%
SYL1657	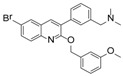	0.16%	25.76%	89.23%	93.92%
SYL1658	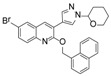	36.88%	19.23%	77.41%	72.67%
SYL1683	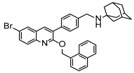	21.73%	26.27%	95.73%	104.43%
SYL1711	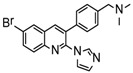	38.33%	25.47%	98.65%	90.69%
SYL1712	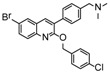	5.75%	55.35%	99.39%	96.93%

**Table 2:** CC_50_ (A549), IC_50_ (anti-HIV-1/EBOV) and SI values of 10 selected quinoline compounds.

ID	IC_50_ (μM)	CC_50_ (μM)	SI
SYL1640	2.96	190.4	64.3
SYL1642	5.21	152.2	29.2
SYL1654	4.98	222.5	44.7
SYL1655	2.65	132.3	49.9
SYL1657	3.56	214.6	60.3
SYL1658	8.65	109.5	12.7
SYL1660	2.58	184.7	71.6
SYL1683	2.93	235.4	80.3
SYL1711	4.11	241.9	58.9
SYL1712	0.95	214.6	225.9

## Supplementary Materials

The following are available online at http://www.mdpi.com/1999-4915/10/12/678/s1. Table S1: Screen scores of Diaryl-Quinoline Compounds.
