# Supplementary material for: Identification of Diaryl-Quinoline Compounds as Entry Inhibitors of Ebola Virus
Source: Viruses. 2018 Nov 30;10(12):678. doi: 10.3390/v10120678 (PMC6315506; doi:10.3390/v10120678)
Supplement: Supplementary file 1 [file viruses-10-00678-s001.pdf]

Number	Structure	Inhibition			Cell Viability
		HIV/H5N1	HIV/LASV	HIV/EBOV	
SYL1619	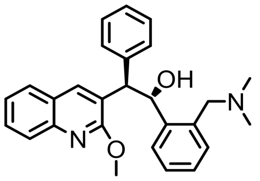	89.75%	88.30%	97.61%	58.03%
SYL1620	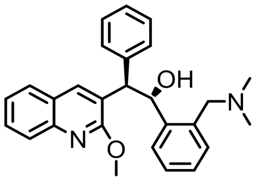	97.47%	80.55%	99.67%	36.50%
SYL1621	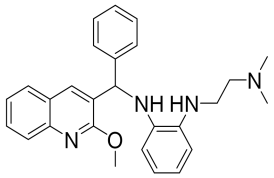	97.96%	99.14%	99.90%	52.36%
SYL1622	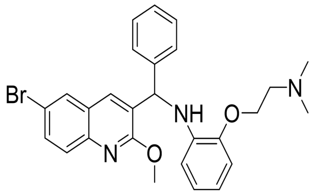	96.99%	83.38%	99.12%	77.49%
SYL1623	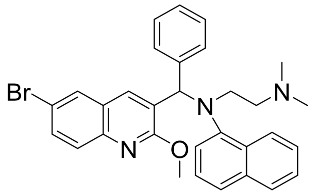	105.28%	15.39%	97.68%	87.19%
SYL1624	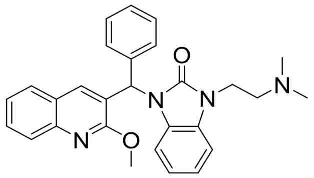	53.54%	85.88%	99.82%	86.63%
SYL1625	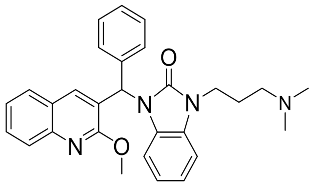	-0.36%	87.77%	99.90%	86.95%
SYL1626	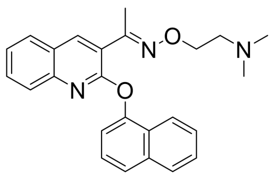	44.19%	85.39%	99.66%	86.91%

SYL1627	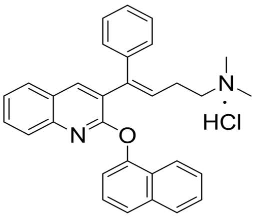	49.35%	87.33%	99.84%	89.09%
SYL1628	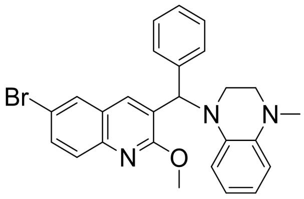	-5.51%	86.50%	99.91%	86.44%
SYL1629	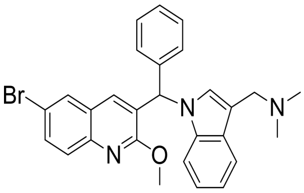	29.75%	88.22%	99.91%	90.62%
SYL1630	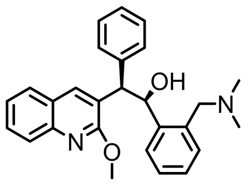	70.34%	91.52%	99.94%	81.98%
SYL1631	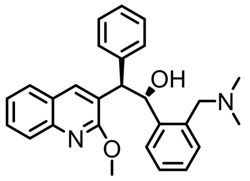	93.68%	92.78%	99.87%	84.50%
SYL1632	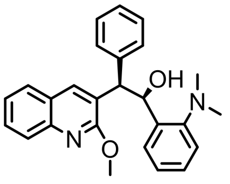	91.83%	90.81%	99.85%	84.20%
SYL1633	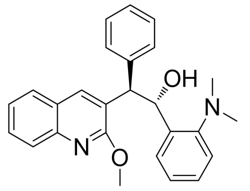	65.18%	84.07%	99.85%	86.77%
SYL1634	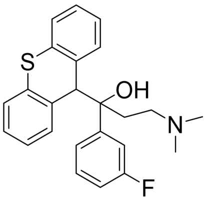	83.95%	83.87%	99.53%	81.21%

SYL1635	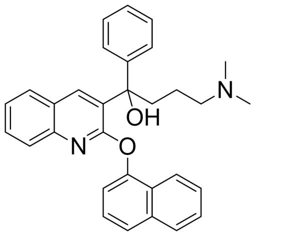	97.52%	99.31%	99.63%	11.15%
SYL1636	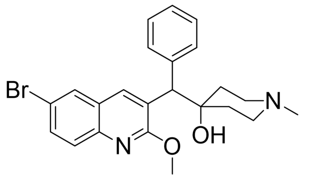	97.70%	98.04%	99.39%	4.08%
SYL1637	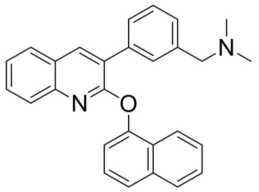	97.92%	81.65%	99.81%	80.11%
SYL1638	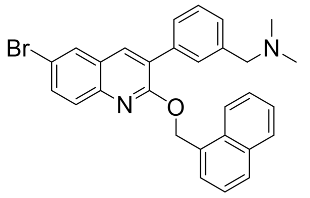	98.27%	99.18%	99.91%	18.62%
SYL1639	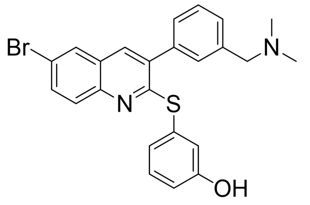	97.65%	98.93%	99.85%	2.71%
SYL1640	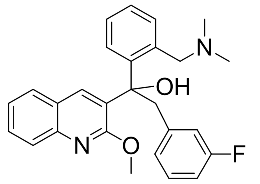	-1.97%	-15.08%	79.06%	97.64%
SYL1641	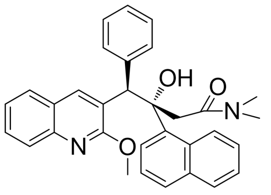	-18.60%	24.99%	64.41%	71.39%
SYL1642	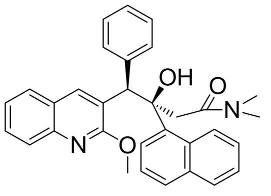	14.11%	34.99%	92.15%	72.30%

SYL1643	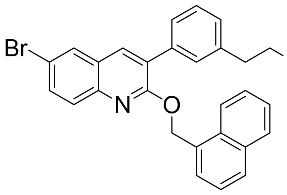	72.74%	83.87%	99.82%	85.39%
SYL1644	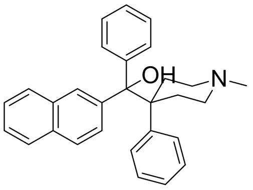	97.63%	92.36%	99.92%	78.35%
SYL1645	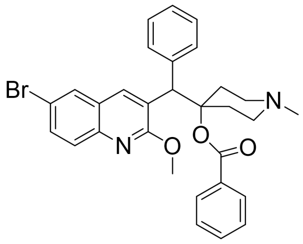	45.37%	86.68%	99.92%	82.47%
SYL1646	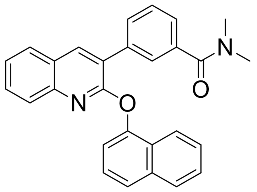	-23.29%	82.50%	99.92%	83.55%
SYL1647	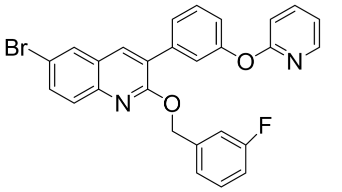	44.09%	89.08%	99.94%	82.66%
SYL1648	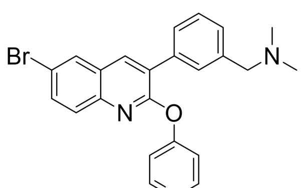	37.82%	89.27%	99.91%	84.21%
SYL1649	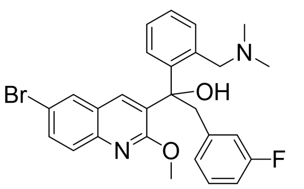	79.45%	90.46%	99.90%	81.62%
SYL1650	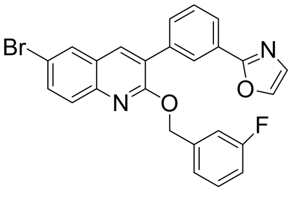	-15.72%	87.63%	99.92%	81.60%

SYL1651	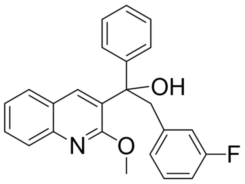	30.01%	89.23%	99.95%	79.56%
SYL1652	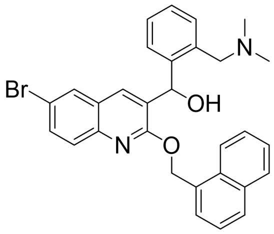	98.16%	97.16%	99.94%	61.30%
SYL1653	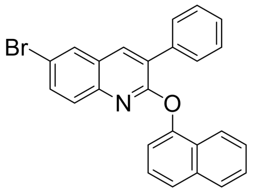	64.36%	87.04%	99.85%	82.57%
SYL1654	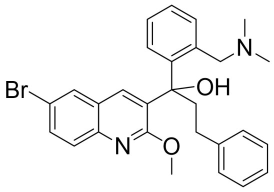	8.04%	18.79%	84.84%	94.74%
SYL1655	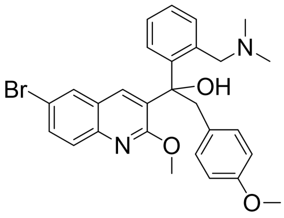	-17.17%	28.87%	90.29%	95.31%
SYL1656	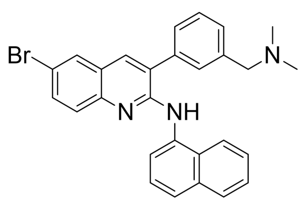	49.96%	51.43%	79.70%	77.79%
SYL1657	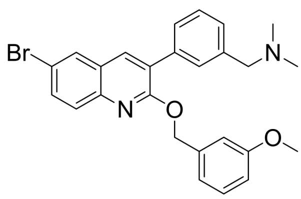	0.16%	25.76%	89.23%	93.92%
SYL1658	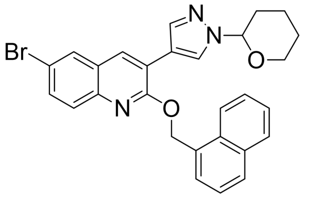	36.88%	19.23%	77.41%	72.67%

SYL1659	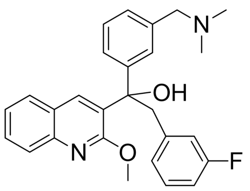	96.52%	97.39%	99.09%	36.09%
SYL1660	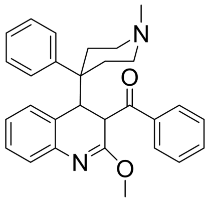	3.34%	-26.55%	97.48%	106.02%
SYL1661	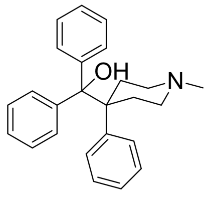	5.09%	72.11%	99.91%	77.89%
SYL1662	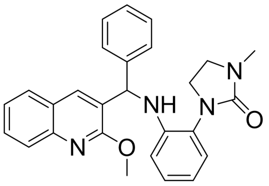	68.52%	89.05%	99.56%	89.06%
SYL1663	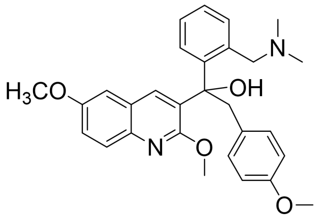	88.29%	89.19%	99.90%	89.15%
SYL1664	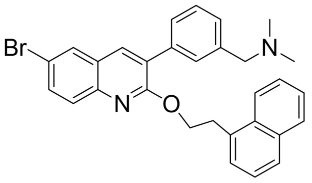	50.44%	86.44%	99.90%	84.59%
SYL1665	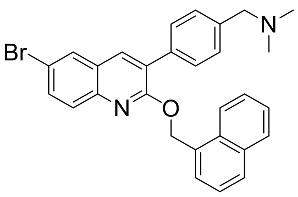	84.24%	88.72%	99.92%	82.87%
SYL1666	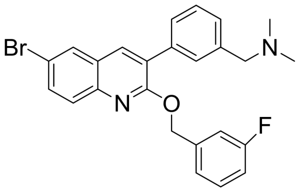	87.06%	91.03%	99.92%	81.28%

SYL1667	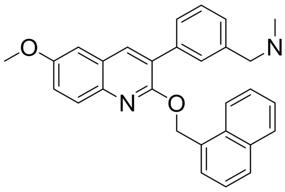	85.49%	85.91%	99.94%	83.17%
SYL1668	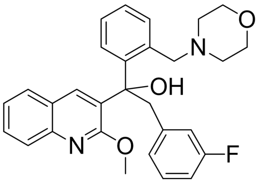	74.86%	86.46%	99.87%	79.06%
SYL1669	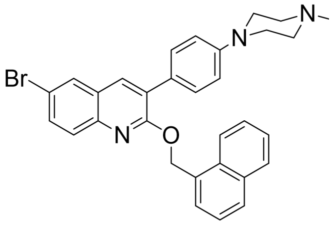	96.42%	92.49%	99.94%	82.47%
SYL1670	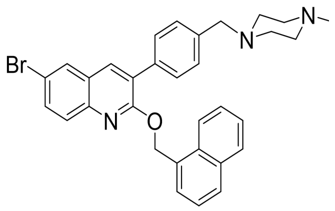	91.10%	77.77%	99.92%	82.37%
SYL1671	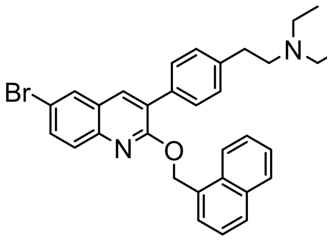	92.36%	89.90%	99.87%	88.97%
SYL1672	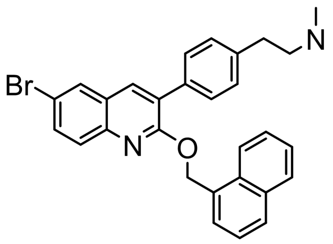	98.01%	99.22%	99.85%	2.06%

SYL1673	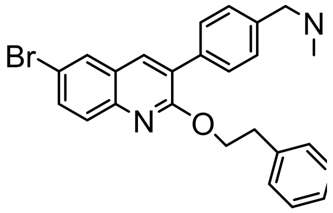	97.21%	94.93%	99.57%	88.82%
SYL1675	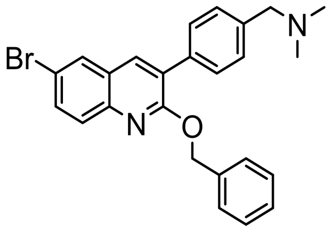	98.13%	91.37%	99.89%	88.37%
SYL1676	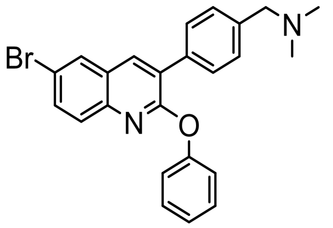	36.23%	19.98%	71.19%	89.65%
SYL1677	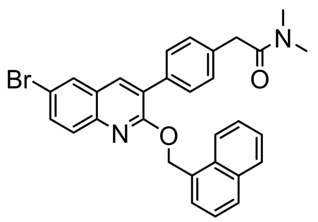	15.95%	29.51%	90.04%	58.71%
SYL1678	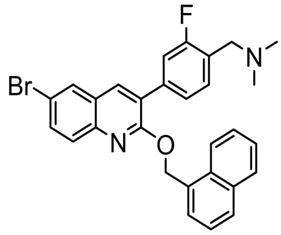	93.90%	97.25%	99.38%	122.25%
SYL1679	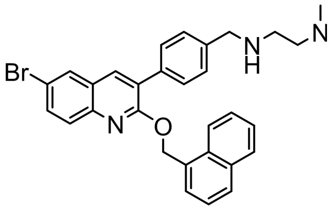	96.95%	97.63%	99.70%	117.07%

SYL1682	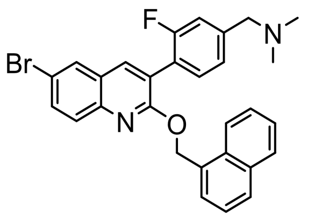	78.74%	94.69%	99.91%	84.09%
SYL1683	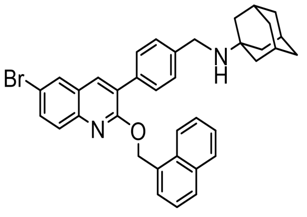	21.73%	26.27%	95.73%	104.43%
SYL1685	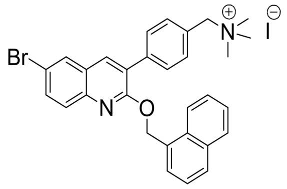	65.25%	93.29%	99.90%	103.42%
SYL1686	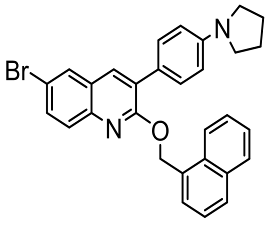	65.78%	90.35%	99.92%	82.03%
SYL1687	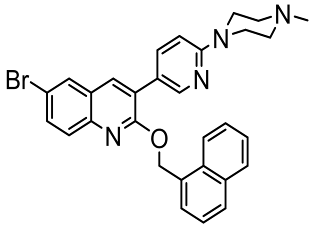	31.90%	88.94%	99.94%	78.11%
SYL1688	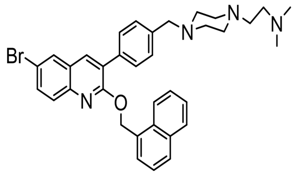	91.36%	88.12%	99.92%	80.00%
SYL1689	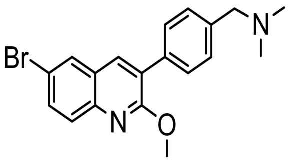	97.51%	88.23%	99.94%	78.79%

Exact Mass:

SYL1690	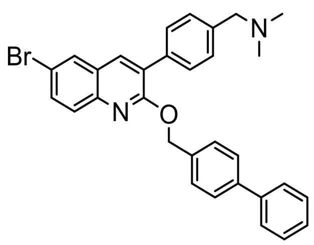	98.15%	86.95%	99.85%	80.30%
SYL1691	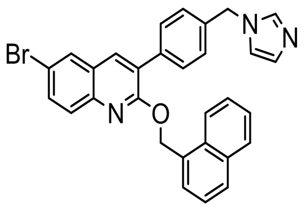	97.25%	57.43%	92.17%	72.14%
SYL1692	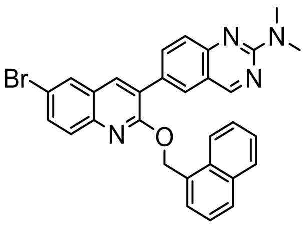	-2.23%	11.86%	48.65%	87.98%
SYL1693	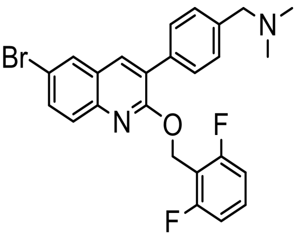	97.66%	99.04%	99.59%	3.19%
SYL1696	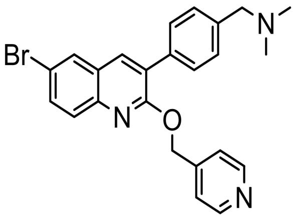	97.45%	98.53%	99.25%	26.82%
SYL1697	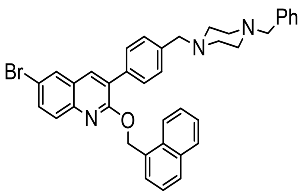	-5.87%	15.89%	67.57%	90.86%
SYL1698	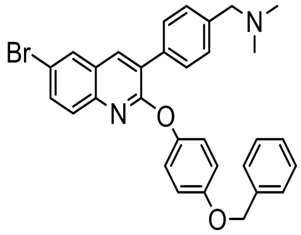	97.44%	98.80%	99.64%	107.16%

SYL1699	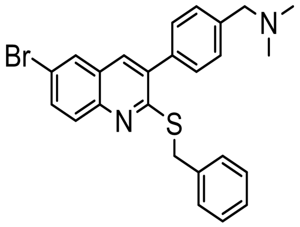	94.56%	93.73%	99.94%	105.32%
SYL1701	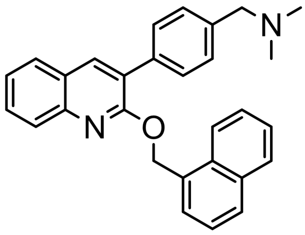	98.51%	97.96%	99.94%	88.01%
SYL1702	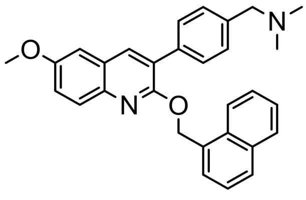	87.31%	89.93%	99.95%	89.90%
SYL1705	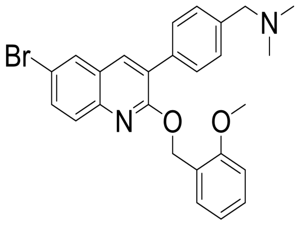	59.98%	88.92%	99.76%	86.34%
SYL1706	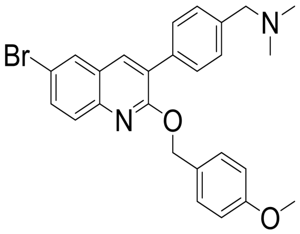	54.58%	87.40%	99.20%	82.99%
SYL1707	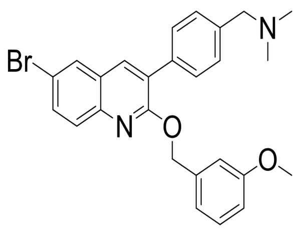	95.43%	78.74%	99.14%	80.79%
SYL1708	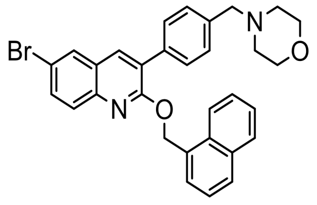	18.60%	82.96%	98.58%	80.29%

SYL1709	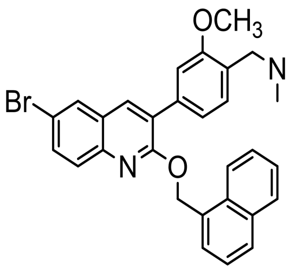	94.56%	81.68%	99.76%	74.44%
SYL1710	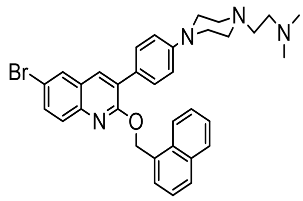	97.63%	98.64%	99.39%	1.97%
SYL1711	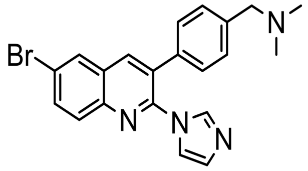	38.33%	25.47%	98.65%	90.69%
SYL1712	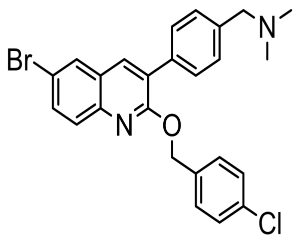	5.75%	55.35%	99.39%	96.93%
SYL1713	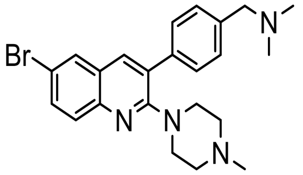	97.68%	99.40%	99.58%	1.24%
SYL1714	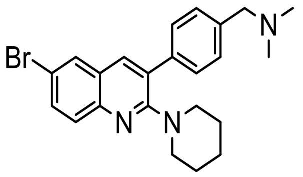	83.36%	51.47%	98.67%	91.09%
SYL1715	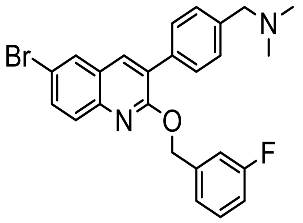	95.43%	76.84%	99.61%	84.72%

SYL1716	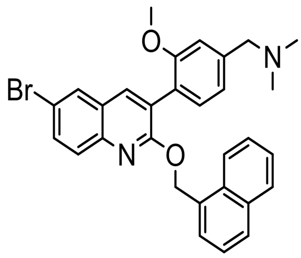	98.20%	99.34%	99.70%	1.73%
SYL1721	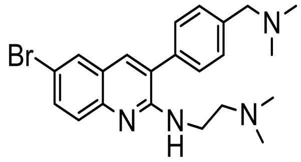	98.20%	99.50%	99.63%	1.26%
SYL1723	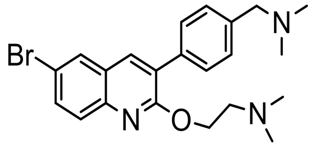	98.10%	97.12%	99.81%	68.89%
